# Evaluation of Different Decellularization Protocols for Obtaining and Characterizing Canine Cardiac Extracellular Matrix

**DOI:** 10.3390/biomedicines12061190

**Published:** 2024-05-27

**Authors:** Izabela Gabriela Rodrigues da Silva, Maria Angelica Miglino, Samara Silva de Souza, Daniela Vieira Buchaim, Rogerio Leone Buchaim

**Affiliations:** 1 Graduate Program in Anatomy of Domestic and Wild Animals, Faculty of Veterinary Medicine and Animal Science, University of Sao Paulo (FMVZ-USP), Sao Paulo 05508-270, Brazil; izabelarodrigues@usp.br (I.G.R.d.S.); miglino@usp.br (M.A.M.); danibuchaim@alumni.usp.br (D.V.B.); 2 Postgraduate Program in Structural and Functional Interactions in Rehabilitation, University of Marilia (UNIMAR), Marilia 17525-902, Brazil; 3 Postgraduate Program in Animal Health, Production and Environment, University of Marilia (UNIMAR), Marilia 17525-902, Brazil; 4 Graduate Program in Biotechnology (PPGBIOTEC), Federal Technological University of Parana (UTFPR), Campus Dois Vizinhos, Dois Vizinhos 85660-000, Brazil; samarasouza@utfpr.edu.br; 5 Medical School, University Center of Adamantina (UNIFAI), Adamantina 17800-000, Brazil; 6 Department of Biological Sciences, Bauru School of Dentistry (FOB-USP), University of Sao Paulo, Bauru 17012-901, Brazil

**Keywords:** extracellular matrix, decellularization, myocardium, scaffolds, canine heart, regenerative medicine, translational science, tissue engineering, transplants

## Abstract

Cardiovascular diseases are considered the leading cause of mortality globally; even with low mortality in dogs, such diseases are described in the same way in companion animals and humans. This study aimed to devise an effective decellularization protocol for the canine myocardium through the association of physical, chemical, and enzymatic methods, assessing resultant alterations in the myocardial extracellular matrix to obtain a suitable scaffold. Two canine hearts were collected; the samples were sectioned into ±1 cm^2^ fragments, washed in distilled water and 1× PBS solution, and followed by treatment under four distinct decellularization protocols. Sodium Dodecyl Sulfate (SDS) 1% 7 days + Triton X-100 1% for 48 h (Protocol I); Sodium Dodecyl Sulfate (SDS) 1% 5 days + Triton X-100 1% for 48 h (Protocol II); Trypsin 0.05% for 1 h at 36 °C + freezing −80 °C overnight + Sodium Dodecyl Sulfate (SDS) 1% for 3 days, Triton-X-100 for 48 h hours (Protocol III); 0.05% trypsin for 1 h at 36 °C + freezing at −80 °C overnight + 1% Sodium Dodecyl Sulfate (SDS) for 2 days + 1% Triton-X-100 for 24 h (Protocol IV). After analysis, Protocols I and II showed the removal of cellular content and preservation of extracellular matrix (ECM) contents, unlike Protocols III and IV, which retracted the ECM and removed essential elements of the matrix. In theory, although Protocols I and II have similar results, Protocol II stands out for the preservation of the architecture and components of the extracellular matrix, along with reduced exposure time to reagents, making it the recommended protocol for the development of a canine myocardial scaffold.

## 1. Introduction

According to the World Health Organization (WHO), cardiovascular diseases stand out as the primary cause of mortality among chronic non-communicable ailments worldwide. In 2015, WHO reported an estimated 17.7 million deaths attributable to cardiovascular diseases, which accounted for 31% of global mortality. Among cardiovascular diseases, acute myocardial infarction is the leading cause of morbidity and mortality. Myocardial infarction is characterized as a disease that generates ventricular remodeling, fibrosis, necrosis, and later heart failure, which can lead to partial or total cardiac dysfunction of the ventricles [1,2,3,4]. Despite the heart’s limited regenerative capacity in adults, it exhibits remarkable reparative abilities. Mortality is linked to long-term effects due to the coordinated deposition of a dynamic extracellular matrix network [5].

There are several therapeutic approaches for the treatment of cardiovascular diseases, but transplantation remains the gold standard for the cure of end-stage heart failure. However, transplantation is limited due to the high cost of the operation, the possibility of rejection, the need for long-term treatment with autoimmune drugs, the limitation of cell therapy, and the shortage of donors. The shortfall in donor organs has been aggravated after the 2020 pandemic, which resulted in a 16% drop in the total number of transplants performed worldwide and a 29% reduction in Brazil [1,4,6]. Such adversities make scientists consider tissue engineering as an alternative treatment method [7]. Although there are fewer data on myocardial infarction and cardiac ischemia in veterinary medicine when compared to human medicine, cardiomyopathies are described in a similar way as a cause of sudden death in companion animals [8,9].

Over the past 25 years, regenerative medicine and tissue engineering have evolved considerably. The knowledge in this field of research has progressed from the ability to recreate functional and physiological tissues in vitro to the recreation of entire systems and organs. Despite tissue engineering being a relatively new area, there is great promise to revolutionize regenerative medicine [10]. Based on this, cardiac tissue engineering emerges as a particularly promising branch, encompassing a diverse array of biomedical, biotechnological, and engineering techniques. These techniques leverage a combination of cells, biological and/or synthetic materials, growth factors, differentiation factors, pro-angiogenic factors, and a monitoring system to induce the regeneration or replacement of the heart [11]. Regeneration is characterized by the combination of several synergistic processes that act not only to threshold an injury but also to generate new cells to replace the loss of tissue; such a process varies greatly from animal to animal. The mammalian heart lacks a regenerative response after an injury, which differs from some species studied. However, variations in regenerative potential are observed not only between species but also within different life stages. A newborn pig has a regenerative window in the first 2 days after birth, while a mouse can regenerate amputation or infarction lesions during the first 7 days of life. There are also clinical reports of human newborns recovering from myocardial infarction to varying degrees [12,13,14,15,16].

The pioneering work by [17] on the decellularization of rat hearts via coronary artery perfusion leveraged the process of obtaining whole-organ cardiac scaffolds. Scaffolds are fundamental to tissue engineering, providing three-dimensional porous structures crucial for tissue restoration. They offer vital support for cellular growth, proliferation, and the formation of new tissues, as well as playing a considerable role in drug delivery [7,10].

Extracellular matrix from decellularized tissues and organs has been used for years in regenerative medicine and tissue engineering. The matrix decellularization process can be carried out by means of physical, chemical, and/or enzymatic processes, which include freeze and thaw cycles, the use of proteolytic enzymes and ionic and anionic detergents, and the use of the stirring or static method [18,19]. After the decellularization process, both the structural integrity and chemical composition of the decellularized tissue are preserved, which gives the matrix a promising cell culture capacity. At first, decellularization efforts primarily focused on cultivating stem or parenchymal cells for tissue regeneration or organ recreation. However, recently, there has been a change in interest; there are several studies directing the use of the decellularized extracellular matrix as powder and hydrogel for organ repair and regeneration [19].

In this sense, knowing the pandemic factor of cardiovascular diseases and the practically null capacity for cardiac tissue regeneration, with the dog as an experimental model, we sought to characterize and standardize a protocol for decellularization of canine myocardium through the association of physical, chemical, and enzymatic methods and to verify if there is complete removal of the cellular content and preservation of ECM components. To date, there are no reports in the literature related to the decellularization and characterization of the extracellular matrix of the canine myocardium.

## 2. Materials and Methods

### 2.1. Animals

The experiment was conducted at the Department of Surgery of the Faculty of Veterinary Medicine and Zootechny (FMVZ/USP), in the discipline of anatomy. Two hearts from canine carcasses were used for the experiment (Table 1). For the qualitative and quantitative evaluations, 6 fragments of each heart were analyzed for each protocol and 6 fragments for the control (native).

### 2.2. Decellularization Process

**Figure 1:**
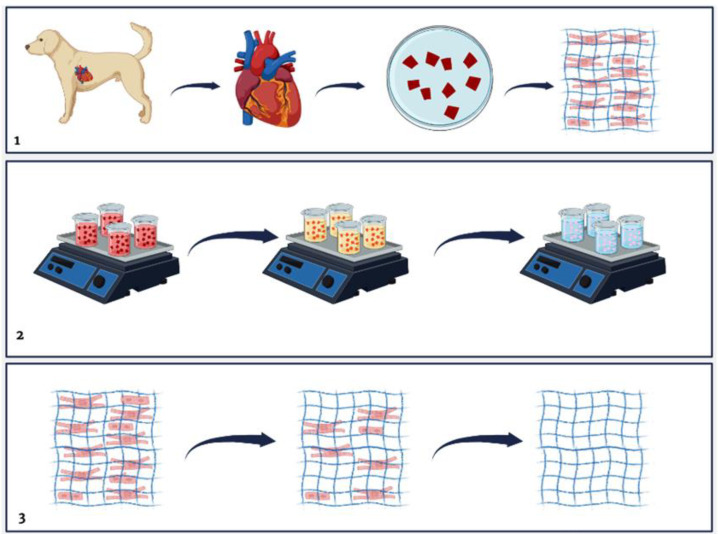
Schematic representation of the decellularization process. (**1**) The heart was collected, and the left and right ventricles were sectioned in ±1 cm^2^. The fragments were divided into four groups and washed in distilled water and PBS 1× for 60 min each at 100 rpm; (**2**) After two hours, the fragments were washed in four different protocols combining detergents and enzymes: Sodium Dodecyl Sulfate (SDS), Triton X-100, and Trypsin at different time intervals. They were then washed in PBS 1× for 48 h; (**3**) Representation of the ECM before, during, and after the decellularization process. Created on Biorender.com.

The decellularization process was based on the use of physical and chemical methods. Mechanical agitation (100 rpm) was used with the submerged material. Initially, the myocardial walls of the left and right ventricles were selected for use in the decellularization process, where they were sectioned into ±1 cm^2^ and later into ±0.5 cm and divided into the left ventricle (LV) and right ventricle (RV) (Figure 1) and subdivided into four groups. Subsequently, they went through a process of two washes lasting 30 min in distilled water; then, the material was washed in a 1× PBS solution for about 60 min. The decellularization process was based on four protocols.

Protocol I was performed with the tissue (LV and RV) kept at room temperature and by immersion in Sodium Dodecyl Sulfate (SDS # SDS, #13-1313-01, LGCBio, Cotia, Brazil) at 1% for 7 days; wash in overnight distilled water, Triton-X-100 (#13-1315-05, LGCBio) at 1% for 48 h. In Protocol II, the material was kept at room temperature and immersed in 1% Sodium Dodecyl Sulfate (#SDS, #13-1313-01, LGCBio) for 5 days, then washed in overnight distilled water and Triton X-100 (#13-1315-05, LGCBio) at 1% for 48 h. Protocol III started with the immersion of the material in 0.05% Trypsin for 1 h at 36 °C, freezing at −80 °C overnight, immersion in Sodium Dodecyl Sulfate (SDS # SDS, #13-1313-01, LGCBio) at 1% for 3 days, washing in distilled water overnight, and Triton X-100 (#13-1315-05, LGCBio) for 48 h. In Protocol IV, the material was immersed in 0.05% Trypsin for 1 h at 36 °C, then subjected to freezing at −80 °C overnight, immersion in Sodium Dodecyl Sulfate (#SDS, #13-1313-01, LGCBio) at 1% for 2 days, washing in distilled water overnight, and Triton X-100 (#13-1315-05, LGCBio) at 1% for 24 h.

### 2.3. Qualitative Histological Analysis

For histological analysis, both native tissue samples and decellularized samples were fixed in 4% paraformaldehyde (PFA) for 48 h, dehydrated in an increasing series of alcohols (70%, 80%, 90%, and 100%), diaphanized in xylol I and II, and embedded in paraffin. Sections of 5 μm, performed in a microtome (RM2265, Leica Biosystems^®^, Wetzlar, Germany), were transferred to glass slides and stained by Hematoxylin and Eosin (HE), Alcian Blue (AB), Masson’s Trichrome (TM), and Picrosirius-red (PR) stains. After staining, the slides were evaluated under light microscopy (Nikon Eclipse 80^®^, Tokyo, Japan).

### 2.4. Quantification of Glycosaminoglycans (GAG) and Collagen

In order to quantify GAG and collagen within the myocardium samples, both native and decellularized tissues were stained with the Alcian Blue pH 2.5 Histokit (EP-12-20018) and Masson’s Trichrome and subsequently photodocumented. The concentrations of GAGs and collagen were estimated using the color deconvolution method through the plugin Color Decovolution2 installed in the ImageJ 1.53e software.

### 2.5. Scanning Electron Microscopy (SEM)

For ultrastructure analysis, the samples were fixed in Carnovsky solution, followed by ultrasound-washed, treated in alcohol crescent solution, dehydrated at a critical point (Leica EM CPD300^®^, Leica Microsystems, Wetzlar, Germany), covered in gold in a metallizer (Emitech Ltd.^®^, Ahford, Kent, UK), and analyzed under a scanning electron microscope (LEO 435 VP; LEO Electron Microscopy Ltd.^®^, Cambridge, UK). Scanning electron microscopy analysis was performed to evaluate the physical, architectural, and tissue ultrastructure of the native and decellularized samples.

### 2.6. Statistical Analysis of the Quantification of Glycosaminoglycans and Collagen

The collagen and glycosaminoglycan measurements of the decellularized and native heart tissue samples were organized in Excel spreadsheets (Microsoft Office Excel^®^version 2404, Redmond, WA, USA) and subjected to an ordinary one-way ANOVA with multiple comparisons, followed by Tukey’s post-test, using GraphPad Prism version 8.0 (GraphPad^®^ Software, La Jolla, CA, USA). Numerical values demonstrate mean ± standard deviation (SD), and the level of statistical significance was set at *p* < 0.05.

## 3. Results

### 3.1. Qualitative Histological Analysis

**Figure 2:**
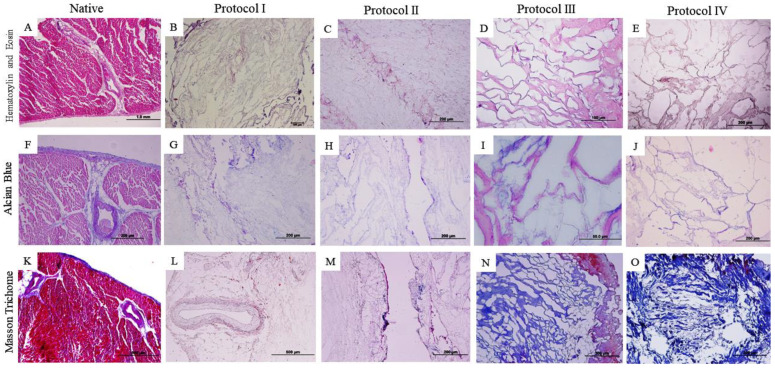
Histological images of the canine myocardium (left ventricle) stained with Hematoxylin and Eosin (**A**–**E**), Alcian Blue (**F**–**J**), and Masson’s Trichrome (**K**–**O**). (**A**,**F**,**K**): native myocardium; (**B**,**G**,**L**): decellularized myocardium using 1% Sodium Dodecyl Sulfate (SDS) for 7 days; 1% Triton-X-100 for 48 h; (**C**,**H**,**M**): 1% Sodium Dodecyl Sulfate (SDS) for 5 days, 1% Triton X-100 for 48 h; (**D**,**I**,**N**): Trypsin 0.05% for 1 h at 36 °C, freeze −80 °C overnight, 1% Sodium Dodecyl Sulfate (SDS) for 3 days, Triton X-100 for 48 h; (**E**,**J**,**O**): Trypsin 0.05% for 1 h at 36 °C, freeze −80 °C overnight, 1% Sodium Dodecyl Sulfate (SDS) for 2 days, 1% Triton X-100 for 24 h.

**Figure 3:**
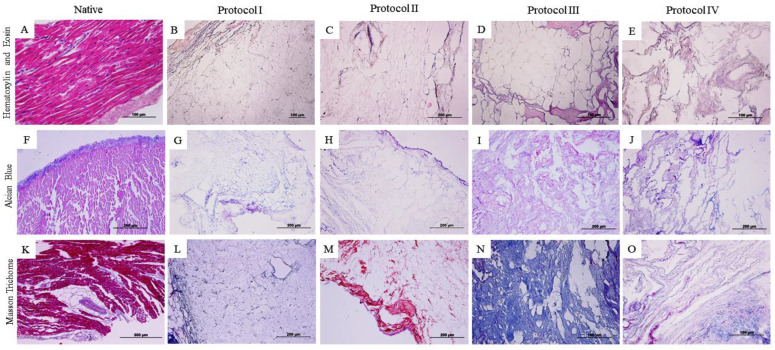
Histological images of the canine myocardium (right ventricle) stained in Hematoxylin and Eosin (**A**–**E**), Alcian Blue (**F**–**J**), and Masson’s Trichrome (**K**–**O**). (**A**,**F**,**K**): native myocardium; (**B**,**G**,**L**): decellularized myocardium using 1% Sodium Dodecyl Sulfate (SDS) for 7 days; 1% Triton-X-100 for 48 h; (**C**,**H**,**M**): 1% Sodium Dodecyl Sulfate (SDS) for 5 days, 1% Triton X-100 for 48 h; (**D**,**I**,**N**): Trypsin 0.05% for 1 h at 36 °C, freeze −80 °C overnight, 1% Sodium Dodecyl Sulfate (SDS) for 3 days, Triton X-100 for 48 h; (**E**,**J**,**O**): Trypsin 0.05% for 1 h at 36 °C, freeze −80 °C overnight, 1% Sodium Dodecyl Sulfate (SDS) for 2 days, 1% Triton X-100 for 24 h.

Histological analysis demonstrates that the applied decellularization protocols resulted in a cell-free scaffold, as can be seen in the staining of Hematoxylin and Eosin (HE). However, it is noted that in Protocols (III and IV) based on the use of the trypsin enzyme associated with SDS and Triton X-100, the architecture of the scaffold differs from the others due to distortion and contracture of the extracellular matrix (Figure 2 and Figure 3).

Alcian Blue (F–J) staining was applied to visualize glycosaminoglycans (GAG) highlighted in blue. In the analysis in question, the samples from the myocardial wall of the left ventricle (Figure 2I,J) and the right ventricle (Figure 3I) do not show evident glycosaminoglycan staining when compared to the other samples.

Masson’s trichrome staining (Figure 3K–O) demonstrates the preservation of collagen fibers (stained in blue) in both treated ventricles, with the exception of the right ventricle sample (Figure 3M) treated with Protocol II (SDS 1% for 5 days and Triton X-100 1% for 48 h), which differs its staining from the others, evidencing the non-preservation of collagen fibers.

**Figure 4:**
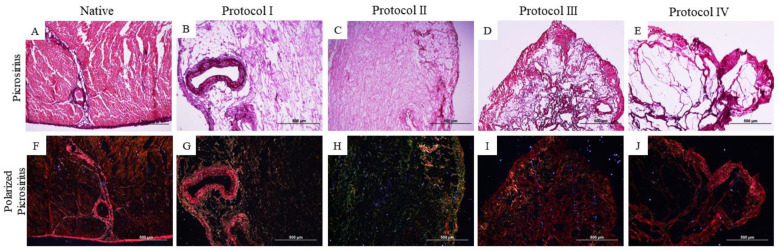
Histological images of the canine myocardium (left ventricle) stained with non-polarized Picrosirius-red (**A**–**E**) and polarized Picrosirius-red (**F**–**J**). (**A**,**F**): Native myocardium; (**B**,**G**): Decellularized myocardium using 1% Sodium Dodecyl Sulfate (SDS) for 7 days, 1% Triton-X-100 for 48 h; (**C**,**H**): 1% Sodium Dodecyl Sulfate (SDS) for 5 days, 1% Triton X-100 for 48 h; (**D**,**I**): Trypsin 0.05% for 1 h at 36 °C, freeze −80 °C overnight, 1% Sodium Dodecyl Sulfate (SDS) for 3 days, Triton X-100 for 48 h; (**E**,**J**): Trypsin 0.05% for 1 h at 36 °C, freeze −80 °C overnight, 1% Sodium Dodecyl Sulfate (SDS) for 2 days, 1% Triton X-100 for 24 h.

**Figure 5:**
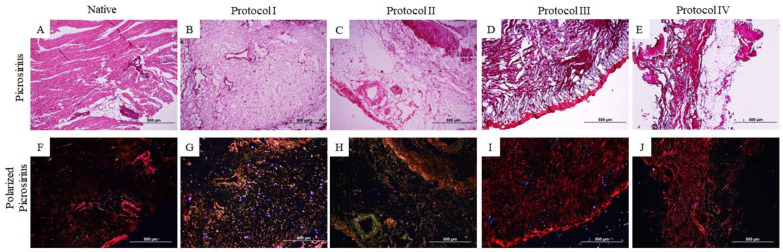
Histological images of the canine myocardium (right ventricle) stained with non-polarized Picrosirius-red (**A**–**E**) and polarized Picrosirius-red (**F**–**J**). (**A**,**F**): Native myocardium; (**B**,**G**): Decellularized myocardium using 1% Sodium Dodecyl Sulfate (SDS) for 7 days, 1% Triton-X-100 for 48 h; (**C**,**H**): 1% Sodium Dodecyl Sulfate (SDS) for 5 days, 1% Triton X-100 for 48 h; (**D**,**I**): Trypsin 0.05% for 1 h at 36 °C, freeze −80 °C overnight, 1% Sodium Dodecyl Sulfate (SDS) for 3 days, Triton X-100 for 48 h; (**E**,**J**): Trypsin 0.05% for 1 h at 36 °C, freeze −80 °C overnight, 1% Sodium Dodecyl Sulfate (SDS) for 2 days, 1% Triton X-100 for 24 h.

Further characterization of collagen types was performed using Picrosirius-red staining (Figure 4 and Figure 5). This revealed the conservation of different types of collagen (Figure 4F–J and Figure 5F–J) through the red–yellow colors (corresponding to the presence of collagen I/thick fibers) and the green color (corresponding to the presence of collagen III/thin fibers).

### 3.2. Scanning Electron Microscopy (SEM)

**Figure 6:**
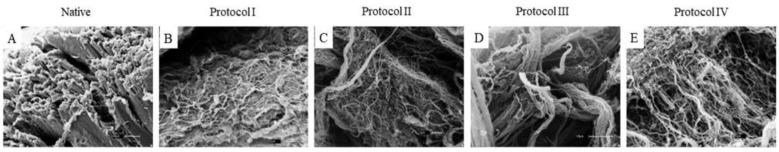
Scanning Electron Microscopy: Left Ventricle. (**A**): Native myocardium; (**B**): decellularized myocardium using 1% Sodium Dodecyl Sulfate (SDS) for 7 days, 1% Triton-X-100 for 48 h; (**C**): 1% Sodium Dodecyl Sulfate (SDS) for 5 days, 1% Triton X-100 for 48 h; (**D**): Trypsin 0.05% for 1 h at 36 °C, freeze overnight at −80 °C, 1% Sodium Dodecyl Sulfate (SDS) for 3 days, Triton X-100 for 48 h; (**E**): Trypsin 0.05% for 1 h at 36 °C, freeze overnight at −80 °C, 1% Sodium Dodecyl Sulfate (SDS) for 2 days, 1% Triton X-100 for 24 h.

**Figure 7:**
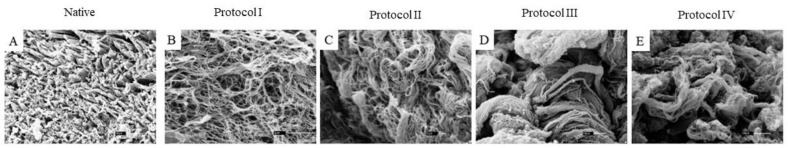
Scanning Electron Microscopy: Right Ventricle. (**A**): Native myocardium; (**B**): decellularized myocardium using 1% Sodium Dodecyl Sulfate (SDS) for 7 days, 1% Triton-X-100 for 48 h; (**C**): 1% Sodium Dodecyl Sulfate (SDS) for 5 days, 1% Triton X-100 for 48 h; (**D**): Trypsin 0.05% for 1 h at 36 °C, freeze overnight at −80 °C, 1% Sodium Dodecyl Sulfate (SDS) for 3 days, Triton X-100 for 48 h; (**E**): Trypsin 0.05% for 1 h at 36 °C, freeze overnight at −80 °C, 1% Sodium Dodecyl Sulfate (SDS) for 2 days, 1% Triton X-100 for 24 h.

The ultrastructure of the native and decellularized tissues was verified by means of SEM, where it was possible to observe the cell removal in the four protocols used. Notably, the composition of the fibers (webs and spirals) and the orientation of the myocardial ECM were preserved while the cardiac cells were removed. However, the difference in the preservation of the ultrastructure of the fibers in the different groups is notorious. In the native groups (left and right ventricles), it is possible to verify the presence of cells and the division and preservation of muscle fibers in the tissues analyzed (Figure 6 and Figure 7).

Samples treated with Protocols I and II (SDS 1% for 7 days and Triton X-100 1% for 48 h; SDS 1% for 5 days and Triton X-100 1% for 48 h) in both the left and right ventricles (Figure 6B,C and Figure 7B,C) exhibited complete cell removal and preserved various types of collagen fibers, which suggests that the use of 1% SDS associated with 1% Triton X-100 is useful in myocardial tissue. However, it is noticeable that, despite the complete cell removal in both protocols, there is a difference in the architecture and preservation of the different collagen fibers. The ultrastructure of Protocol II evidences the preservation of thick and thin fibers in a higher proportion than in Protocol I. This suggests that although the detergent concentration was identical and the time of exposure to the solution in Protocol II was longer, it did not negatively interfere with matrix preservation.

Samples submitted to enzymatic treatment in Protocols III and VI (Trypsin 0.05%, SDS 1% for 3 days, Triton X-100 1%; Trypsin 0.05%, SDS 1% for 2 days, Triton X-100 1%) demonstrate complete cell removal and preserved different types of collagen, along with architectural integrity (Figure 6D,E and Figure 7D,E). However, as in the histological analysis that differs from the other protocols, the arrangement of collagen fibers (thick and thin) shows a retraction. This is an expected result, due to some results already found in the literature on the mode of cell removal and tissue retraction.

When comparing the protocols employed, both present complete cell removal. However, when analyzing the preservation of thick and thin collagen fibers, Protocol II (Figure 6C and Figure 7C) showed superior preservation of the fibers and architecture. On the other hand, Protocols III and IV (Figure 6D,E and Figure 7D,E), although completely removing the cell content, presented different characteristics in the preservation of the ultrastructure, with a greater presence of thick fibers.

### 3.3. Quantification of Glycosaminoglycans (GAG)

**Figure 8:**
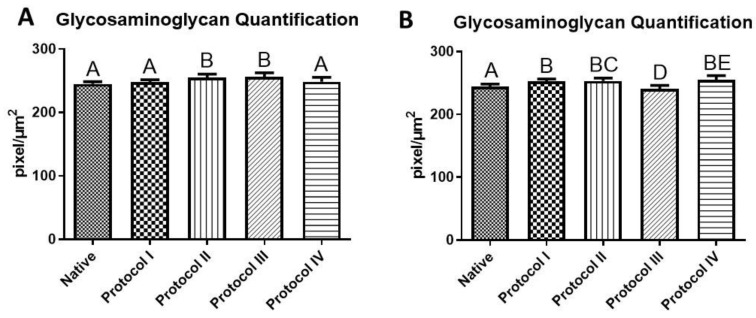
Glycosaminoglycan quantification. Quantification of glycosaminoglycans by the color deconvolution method using the Color_Decovolution2 plugin installed in the ImageJ 1.53e software (https://imagej.nih.gov/ij/index.html; accessed on 2 January 2024). (**A**) Right ventricle; (**B**) Left Ventricle. Protocol I: 1% Sodium Dodecyl Sulfate (SDS) for 7 days and 1% Triton-X-100 for 48 h; Protocol II: 1% Sodium Dodecyl Sulfate (SDS) for 5 days and 1% Triton X-100 for 48 h; Protocol II: 1% Sodium Dodecyl Sulfate (SDS) for 5 days, and 1% Triton X-100 for 48 h; Protocol III: Trypsin 0.05% for 1 h at 36 °C, freeze −80 °C overnight, 1% Sodium Dodecyl Sulfate (SDS) for 3 days, and Triton X-100 for 48 h; Protocol IV: Trypsin 0.05% for 1 h at 36 °C, freeze −80 °C overnight, 1% Sodium Dodecyl Sulfate (SDS) for 2 days, and 1% Triton X-100 for 24 h. Different uppercase letters indicate a significant difference (A ≠ B ≠ C ≠ D ≠ E). Mean ± SD, ANOVA, and Tukey’s post-test, *p* < 0.05.

In addition to verifying the preservation of glycosaminoglycans in all the scaffolds after the decellularization process, quantitatively, it was observed that the amount of glycosaminoglycans in the native tissues (242.6 ± 2.19 pixel/µm^2^) was lower than in the samples treated in the decellularization process, except in Protocol III of the left ventricle (238.4 ± 1.58 pixel/µm^2^) (Figure 8).

In the right ventricle, Protocol II and III scaffolds had the highest averages, with no significant difference between them, but with a difference compared to the others. In the left ventricle, Protocol II had the highest mean (251.8 ± 1.59 pixel/µm^2^), with no significant difference in relation to Protocols I and IV, but with a difference to the native and Protocol III (Figure 8).

### 3.4. Collagen Quantification

**Figure 9:**
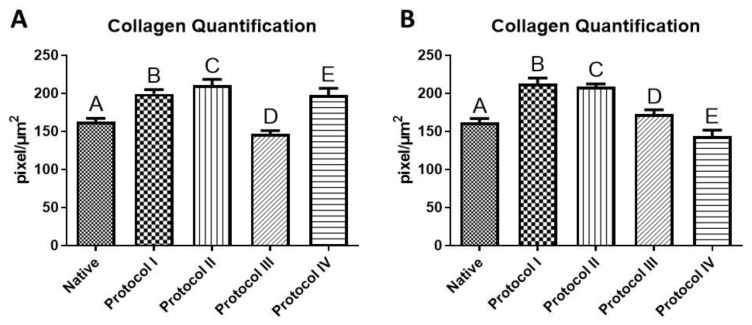
Collagen quantification. Quantification of collagen by the color deconvolution method using the Color_Decovolution2 plugin installed in the ImageJ 1.53e software (https://imagej.nih.gov/ij/index.html; accessed on 2 January 2024). (**A**) Right ventricle; (**B**) Left Ventricle. Protocol I: 1% Sodium Dodecyl Sulfate (SDS) for 7 days, 1% Triton-X-100 for 48 h; Protocol II: 1% Sodium Dodecyl Sulfate (SDS) for 5 days, 1% Triton X-100 for 48 h; Protocol II: 1% Sodium Dodecyl Sulfate (SDS) for 5 days, 1% Triton X-100 for 48 h; Protocol III: Trypsin 0.05% for 1 h at 36 °C, freeze −80 °C overnight, 1% Sodium Dodecyl Sulfate (SDS) for 3 days, and Triton X-100 for 48 h; Protocol IV: Trypsin 0.05% for 1 h at 36 °C, freeze −80 °C overnight, 1% Sodium Dodecyl Sulfate (SDS) for 2 days, 1% Triton X-100 for 24 h. Different uppercase letters indicate a significant difference (A ≠ B ≠ C ≠ D ≠ E). Mean ± SD, ANOVA and Tukey’s post-test, *p* < 0.05.

Collagen quantification showed that the amount of collagen in the protocols used was higher than in native tissue, except in Protocol III of the right ventricle and Protocol IV of the left ventricle. There was a significant difference between all the protocols studied and the native tissue in the two ventricles. The highest mean in the right ventricle was Protocol II, and in the left ventricle, Protocol I (Figure 9).

## 4. Discussion

The high prevalence of cardiovascular diseases requires new therapeutic strategies [20,21]. It is known that cardiovascular diseases are considered the main cause of death among non-communicable diseases, with myocardial infarction being a determining factor for the onset of heart failure. The gold standard treatment for heart failure remains heart transplantation, and tissue engineering within regenerative medicine emerges as a strategic approach to address the challenges within current treatment methods. This involves the development of biological substitutes capable of restoring, improving, or maintaining tissue function. The main objective of decellularization methodologies is to produce scaffolds with the ultrastructure and composition of the native extracellular matrix while removing cellular content and genetic material from tissue [22,23,24]. Several decellularization protocols have been described for almost all tissues and organs of the body and include physical, chemical, and/or enzymatic approaches. It seeks to produce a scaffold that can mimic the characteristics of the myocardium, allowing engineered tissue to provide and improve cell survival and proliferation; although scaffolds have been studied, they have not been able to mimic the architecture and function of native tissue [25,26].

The process of decellularization of the heart tissue or the entire heart is usually based on the use of ionic and nonionic detergents. The whole heart of a rat is decellularized via perfusion [27], slices of the human heart [28], and swine hearts [29]. Using Sodium Dodecyl Sulfate (SDS) resulted in lower DNA content and maintenance of the native structure of the extracellular matrix when compared to approaches using other chemicals. We focus on the search for a scaffold for the canine myocardial ECM, which includes the search for a decellularization protocol and the effects of detergents and enzymes on cardiac tissue. In the current literature, there are several decellularization protocols, but none that use canine myocardium. The methodologies described here generated a decellularized canine myocardial ECM with preservation of the architecture, thus removing cellular contents, maintaining the 3D network, and preserving essential elements of the ECM for cell culture, adhesion, differentiation, and proliferation. The strength of our heart-based protocol was the achievement of a scaffold that enables future studies in regenerative medicine without the need for transplantation [30]. The present work sought to develop a scaffold that can mimic the characteristics of a healthy native myocardial tissue by obtaining an ideal decellularization protocol for the canine myocardium.

None of the decellularization techniques currently used are capable of completely removing the cellular material from a complete tissue or organ, and there is no standard protocol for each tissue since the properties of the tissues change according to the age and origin of the donor species, which makes it necessary to adapt protocols and characterize the matrices [17,26,31,32]. Several studies have shown that SDS and Trinton X-100 are effective in myocardial decellularization, as well as the use of trypsin associated with detergents, but not in the species studied in this study [6,19,31,32,33,34,35,36,37]. Preservation of the biochemical composition of the extracellular matrix is crucial, as the ECM provides bioactive signals that affect the cellular response to its environment, such as proliferation, migration, and differentiation. In this way, to ensure the decellularization of human cardiac tissue while preserving the bioactivity of the extracellular matrix, we optimized the previous methods of decellularization [11,34,38,39,40].

In the histological analysis, Hematoxylin and Eosin staining evidenced the absence of cell contents and nuclei in the four protocols. Respectively, the other stains indicate the preservation of matrix components, such as collagen fibers and glycosaminoglycans, where Protocols I and II (SDS and Triton-100) presented satisfactory results superior to the other protocols used (based on the use of trypsin), due to the preservation of the matrix components and the architecture of the extracellular matrix, results that stand out from the native samples. Previous studies have obtained similar results from the use of SDS and Triton X-100 in samples of different species [41,42,43]. The histological results of protocols I and II show the preservation of the matrix components, which can be highlighted by the quantification of collagen and glycosaminoglycans, as they surpass the data of the native tissue. Both SDS and Triton X-100 protocols preserved type I and type III collagen, as seen in the picrosirius stains, with the demarcation of red and green colors indicative of the qualitative preservation of these matrix components, differently from Protocols III and IV, where red fibers are demarcated. Positive results in the use of SDS and Triton-100 as decellularizing agents, preserving the matrix aspects and its components, have been reported in cardiac tissues of other species in previous studies, with advanced applications of the scaffolds obtained [11,32,35,36,37]. The use of trypsin in Protocols III and IV did not preserve the matrix structure and matrix components as it decellularized the tissue, which is not consistent with previous studies in different species [34,40,41,42,43,44,45].

We know that the use of detergents can alter the structure of the extracellular matrix, as can the use of enzymes such as trypsin. The isolated use of trypsin is little considered due to the high level of degradation of matrix structures and little reduction in cellular content [46]; however, its application with ionic and nonionic detergents produces good scaffolds [19]. It is known that the time of exposure to substances is a crucial factor for tissue preservation; however, there are factors that influence this exposure time, such as the species chosen. In addition to the histological analyses, scanning electron microscopy was performed to evaluate the ultrastructure of the tissue. Collagens are the main structural elements of ECM and are indispensable for the preservation of the mechanical resistance of tissues [47] produced by swine extracellular matrix scaffolds, with excellent results in the preservation of ECM components and ultrastructure, which allowed the use of them in advanced tests for tissue engineering.

In this study, the evaluation of the ultrastructure showed some disorganization in the collagen fibers in Protocols III and IV, adapted from previous work [19], in both ventricles, where the association of Trypsin, SDS, and Triton X-100 generated a rough surface of the matrix ultrastructure with few thin fibers preserved, as described in previous works [34]. Differently from Protocols I and II, despite the long exposure to SDS in Protocol I, the ultrastructure and the thick and less thick collagen fibers remained preserved as much as in Protocol II. Recently, [45] obtained scaffolds of both ventricles using a similar decellularization protocol in porcine samples, creating an extracellular matrix hydrogel. Previous studies with adapted protocols have obtained similar results, which have allowed for crop growth, adhesion, and cell growth [11,34,40].

There is no universal reagent or protocol, but instead, the selection of the most appropriate decellularization approach depends heavily on the end application and will dictate the hierarchy of properties to be retained. The choice is further complicated by variations in composition between tissue types, specifically differences in anatomical structure, function, and age. There are studies based on the use of SDS and Triton X-100 for decellularization of porcine, bovine, rat, and human myocardium; however, due to the factors already mentioned, there is no standard protocol for all tissues. On the other hand, there are also no extensive studies of the decellularized canine cardiac extracellular matrix, although there are reports of myocardial infarction [26,45,46,47,48,49,50].

In our study, the quantitative assessment of glycosaminoglycans and collagen in the 2 ventricles corroborated the qualitative histological and scanning electron microscopy (SEM) results, which showed that Protocol II, followed by Protocol I, was effective in maintaining GAGs and collagen. It is known that GAGs are found in various tissues of the body and have important functions in maintaining the health and integrity of these tissues, which intensify the effects of cytokines, growth factors, and angiogenic factors, allowing cell proliferation and differentiation, as well as favoring angiogenesis [51]. Due to its high biocompatibility and low immunogenicity, collagen is still the protein of choice for preparing biomaterials. The use of collagen as a scaffold has led to the development of bioengineered tissues as substitutes for various tissues [52,53,54].

Despite the promising initial results obtained in our work based on the analyses carried out, there are limitations in our research that can and should be overcome in future studies, such as immunohistochemistry, physicochemical analyses of the scaffolds, matrix sterility testing to enable future cell adhesion assays, and/or the production of a hydrogel. Serving as a basis for studies of the canine cardiac extracellular matrix as a model for the treatment of cardiovascular diseases, both in human medicine and veterinary medicine, in the near future, since the diseases mentioned in the work develop in humans in a similar way in companion animals.

It can also be considered a limitation of this experiment that the number of “dog” animals could have been increased, but we were unable to obtain more hearts from healthy animals. To reduce this limitation, we sampled six fragments for each protocol used (*n* = 24), so our sampling varied within the same organ. Currently, dogs are considered family members in the special health program and cannot be used for studies unless their owners agree to donate their animals’ bodies. Furthermore, it is believed that obtaining and defining the ECM of the dog’s heart would not require the use of multiple hearts, as our focus was not to standardize or systematize the structures of the heart. Our aim was not to standardize or systematize the structures of the heart, but rather to define a suitable and successful procedure for defining the cardiac ECM.

## 5. Conclusions

In conclusion, the present study reports the use of a simple decellularization protocol adapted for canine hearts using the physical decellularization method of mechanical agitation (100 rpm) associated with chemical and enzymatic methods. The resulting scaffolds were cell-free, with key components of the extracellular matrix preserved, such as glycosaminoglycans and collagens. By optimizing the decellularization steps and reducing the SDS exposure time, it is possible to mitigate the negative effects on the essential proteins of the ECM, as observed in Protocol II. The association of the qualitative and quantitative results shows that Protocol II has slightly better results when compared to the other protocols. The simple and time-efficient protocol and preservation of the extracellular matrix make them more suitable for the production of a canine myocardial scaffold. However, further characterization of the matrix and cell adhesion assays is needed to enhance its applicability in tissue engineering, focused on cardiac tissue regeneration.

## Figures and Tables

**Table 1:** Specifications that correlate the animal with breed and age.

Animal	Race	Age
1	Dachshund	1 year
2	NDB	≈14 years old

NDB: No Defined Breed.

## Data Availability

The data presented in this study are available on request from the corresponding author.
